# Significant increase in cyanide degradation by *Bacillus* sp. M01 PTCC 1908 with response surface methodology optimization

**DOI:** 10.1186/s13568-017-0502-2

**Published:** 2017-11-10

**Authors:** Zohre Javaheri Safa, Saeed Aminzadeh, Mohammadreza Zamani, Mostafa Motallebi

**Affiliations:** 1 National Institute for Genetic Engineering and Biotechnology (NIGEB), Shahrak-e Pajoohesh km 15, Tehran-Karaj Highway, Tehran, P. O. Box: 14965/161, Iran; 2 Department of Cellular and Molecular Biology, NourDanesh Institute of Higher Education, Isfahan, Iran

**Keywords:** *Bacillus* sp. M01 PTCC 1908, Biodegradation, Cyanide, One way ANOVA, Plackett–Burman design, Response surface methodology (RSM)

## Abstract

Cyanide is used in many industries despite its toxicity. Cyanide biodegradation is affordable and eco-friendly. Sampling from cyanide-contaminated areas from the Muteh gold mine and isolation of 24 bacteria were performed successfully. The selected bacteria—‘*Bacillus* sp. M01’—showed maximum tolerance (15 mM) to cyanide and deposited in Persian Type Culture Collection by PTCC No.: 1908. In the primary experiments, effective factors were identified through the Plackett–Burman design. In order to attain the maximum degradation by *Bacillus* sp. M01 PTCC 1908, culture conditions were optimized by using response surface methodology. By optimizing the effective factor values and considering the interaction between them, the culture conditions were optimized. The degradation percentage was calculated using one-way ANOVA vs t test, and was found to have increased 2.35 times compared to pre-optimization. In all of the experiments, R^2^ was as high as 91%. The results of this study are strongly significant for cyanide biodegradation. This method enables the bacteria to degrade 86% of 10 mM cyanide in 48 h. This process has been patented in Iranian Intellectual Property Centre under Licence No: 90533.

## Introduction

With the increasing population and development of various industries across the world, soil and groundwater are more prone to contamination than ever before. Cyanide is a carbon–nitrogen radical found in inorganic and organic compounds (Dash et al. 2009). It is very dangerous and toxic. Its toxicity is due to its physicochemical properties (Gurbuz et al. 2009). Because of the cyano group (–C≡N) of cyanide, there are several different forms of cyanide found in nature (Luque-Almagro et al. 2011a; Mirizadeh et al. 2014). Historically, in the First World War, cyanide was used as a chemical weapon (Gupta et al. 2010). Cyanide compounds can be used in gold mining, steel manufacturing, steel making, organic and chemical production, electroplating, polymer synthesis, and other industrial processes, in various industries such as dyes and pharmaceutical, agricultural and resins production, and cassava starch industries; hence, environmental experts also have a concern about it (Adams et al. 2001; Barclay et al. 1998; Glanpracha and Annachhatre 2016; Luque-Almagro et al. 2011b; Potivichayanon and Kitleartpornpairoat 2010; Ricaño-Rodríguez and Lepe 2015). Moreover, cellular catalase and mitochondrial cytochrome oxidase, peroxidase, ascorbic acid oxidase, and other oxidases contain cyanide (Dubey and Holmes 1995; Kao et al. 2003; Raybuck 1992). Cyanide is poisonous for all organisms. Therefore, it is crucial to remove it from industrial effluents (Nallapan Maniyam et al. 2015). In the Philippines, collection of food trades and tropical marine fish for aquarium is carried out by using sodium cyanide (Mak et al. 2005). Large amounts of cyanide are produced regularly by industries in waste water streams; it is an intense health hazard for all living things and ecosystem. Cyanides are frequently removed by biodegradation, chemical and physical methods (Kebeish et al. 2015; Pereira et al. 1996). Biodegradation of cyanide is cheaper than chemical and physical methods and faster than natural oxidation. It is more efficient and has less cost (Gupta et al. 2010; Igeño et al. 2007; Karamba et al. 2015). Chemical treatments used for cyanide degradation are toxic itself when released in ecosystem, expensive and produce some other toxic compounds. Biological treatment is thus the best alternative for cyanide degradation (Luque-Almagro et al. 2011b; Watanabe et al. 1998), because it has fewer side effects compared to other methods and is moreover less expensive (Karamba et al. 2015). Some organisms such as fungi, arthropods, plants, and bacteria can produce cyanide (Khamar et al. 2015). Cyanide produced by fungi and bacteria is used to their advantage by generating the antimicrobial cyanide compounds that inhibit rival organisms (Basile 2008). In this survey, isolation, screening, and biochemical characterization of *Bacillus* sp. M01 PTCC 1908 in the context of significant cyanide degradation capability were studied that can be used in many industries. Isolation and identification of cyanide degrading bacteria are a very important step, and their ability has been studied. Also, cyanide degradation has been optimized by several methods such as Plackett–Burman design and response surface methodology (RSM). Statistical analysis of the model validation was performed by one-way ANOVA vs. t test.

## Materials and methods

### Materials

All materials for this study were purchased from Merck Company. The DNA extraction kit, PCR purification kit, and Master Mix for PCR amplification were bought from Bioneer Company. The 1-Kb DNA Ladder, high pure agarose, and primers were prepared by Massruler™, Invitrogen, and Cinnagen respectively. BeckMan Spectrophotometer DU530 and GFL Shaker Incubator 3031, 3033 were also used.

### Softwares

GraphPad Prism 6 (San Diego, CA, USA) was used to depict the graph. Method designing and data analysis were performed using Minitab 17.1.0 Statistical package (Minitab Inc., State College, PA, USA) and Design Expert 7.0.0 (Stat-Ease, Inc., Minneapolis, MN, USA).

### Samples and culture condition

Bacterial samples were isolated from the wastewater and soil found at the Muteh gold mine (Isfahan-Iran). Bacteria were cultured in nutrient broth medium (pH 6.8) and incubated in a shaking incubator at 37 °C and 180 rpm. All strains were maintained on nutrient agar.

### Screening and bacterial isolation

For screening cyanide-degrading bacteria, 0.5% (V/V) of overnight culture (OD_600_ = 0.86) was inoculated into the nutrient broth medium. Subsequently, different quantities of filter–sterilized KCN were added to determine the degrading ability concentration of cyanide. When the bacteria reached 0.5 McFarland standard turbidity, they were added into the nutrient broth samples containing varying concentration of KCN (1.5, 2.3, and 3.8, 7.6, 11.5, and 15.3 mM). They were then incubated at 180 rpm and 37 °C for 48 h. After this, 1 mL of harvested culture was plated onto nutrient agar. The plate containing the nutrient agar was incubated at 37 °C for 24 h.

### Identification of *Bacillus* sp. M01

The *Bacillus* sp. M01 was identified by analyzing the microbiological and biochemical characteristics of the bacterial colonies.

For molecular identification, the 16S rDNA sequence was studied. The 16S rDNA gene was amplified by PCR using bacterial universal primers 27F (5′AGAGTTTGATCMTGGCTCAG3′) and 1492R (5′TACGGYTACCTTGTTACGACTT3′) (Lane 1991), with an annealing temperature of 50 °C for 30 s and an extension time of 1 min for 30 cycles.

*Bacillus* sp. M01 deposited in Persian Type Culture Collection (PTCC) by PTCC No.: 1908.

### Biodegradation and quantifying the concentration of cyanide by *Bacillus* sp. M01 PTCC 1908

*Bacillus* sp. M01 PTCC 1908 was cultured in nutrient broth medium containing 10 mM of cyanide. After 48 h, the residual cyanide in culture supernatant was measured through picric acid colorimetric assay. Color intensity is correlated with the concentration of cyanide. Absorbance was measured at 492 nm by a UV spectrophotometer. The amount of cyanide consumed by *Bacillus* sp. M01 PTCC 1908 was measured by calculating the absorbance difference between the positive control and the cell-free supernatant (Fisher and Brown 1952; Kandasamy et al. 2015).

### Plackett–Burman design (PBD)

The Plackett–Burman design was used to identify the most important factors in the initial phase of the experiment and to find the key factors for cyanide biodegradation (Liu and Tang 2010; Plackett and Burman 1946). The factors and values is shown in Table 1 in coded form.

**Table 1:** Experimental design using PBD for screening factors and achieved the effective factors on cyanide degradation

Std. order	Variables				
	A	B	C	D	E
1	+ 1	− 1	+ 1	− 1	− 1
2	− 1	+ 1	+ 1	− 1	+ 1
3	− 1	− 1	− 1	+ 1	+ 1
4	+ 1	+ 1	+ 1	− 1	+ 1
5	+ 1	− 1	− 1	− 1	+ 1
6	− 1	+ 1	− 1	− 1	− 1
7	− 1	− 1	− 1	− 1	− 1
8	+ 1	+ 1	− 1	+ 1	− 1
9	+ 1	− 1	+ 1	+ 1	− 1
10	− 1	+ 1	+ 1	+ 1	− 1
11	− 1	− 1	+ 1	+ 1	+ 1
12	+ 1	+ 1	− 1	+ 1	+ 1

A: pH at 5(− 1) and (+ 1) 10, B: temperature at 25(− 1) and (+ 1) 45 °C, C: KCN at 2(− 1) and (+ 1)10 mM, D: shaking speed of 100(− 1) and (+ 1) 200 rpm, E: inoculation amount 2(− 1) and (+ 1) 10

### Central composite design (CCD)

The most effective factors were further optimized by 20-run experimental plan using the CCD method. These factors were studied at five different levels, coded as − α, − 1, 0, + 1 and + α. The screening of factors selected through the Plackett–Burman design is shown in Table 2, in preparation for the CCD optimization step.

**Table 2:** Central composite design by screening the effective factors through the Plackett–Burman design

Coded values	Factors	Range examined	Levels				
			− α	− 1	0	+ 1	+ α
A	Temperature (°C)	25–48	25.2	30	37	44	48.7
B	Rotation speed (rpm)	95–264	96	130	180	230	264
C	Inoculation amount %	1.59–18.4	1.6	5	10	15	18.4

Moreover, the full experimental plan, with regard to their values in coded form, is provided in Table 1. The effect of variables, interaction between them and statistical analysis was performed to achieve the high degradation (Altaf et al. 2006; Dayananda et al. 2005). The equation used in this model is as follows:

1 $$\begin{aligned} {\text{Y}} & = \upbeta_{0} + \upbeta_{1} {\text{X}}_{1} + \upbeta_{2} {\text{X}}_{2} + \upbeta_{3} {\text{X}}_{3} + \upbeta_{4} {\text{X}}_{1}^{2} \hfill \\ & \quad + \upbeta_{5} {\text{X}}_{2}^{2} + \upbeta_{6} {\text{X}}_{1} {\text{X}}_{2} + \upbeta_{7} {\text{X}}_{1} {\text{X}}_{3} + \upbeta_{8} {\text{X}}_{2} {\text{X}}_{3} \hfill \\ \end{aligned}$$

where Y is the final cyanide degradation, β_i_ is the model coefficient, and X_i_ depicts the variables (Modiri et al. 2015).

### Statistical analysis of the model

Finally, a series of experiments was performed on optimized, predicted values that were suggested by the software as the optimized points and pre-optimized conditions in five replicates. Data were analyzed using the one-sample t test and one-way ANOVA method. The P value was greater than 0.05, implying that the three variables have a significant impact on the biodegradation of cyanide.

Molecular characterization, performed by 16S rDNA gene sequencing, confirmed the results of the microbiological and biochemical studies (Genbank Accession Number KR996794).

## Results

### Isolation and screening cyanide-degrading bacteria

**Fig. 1:**
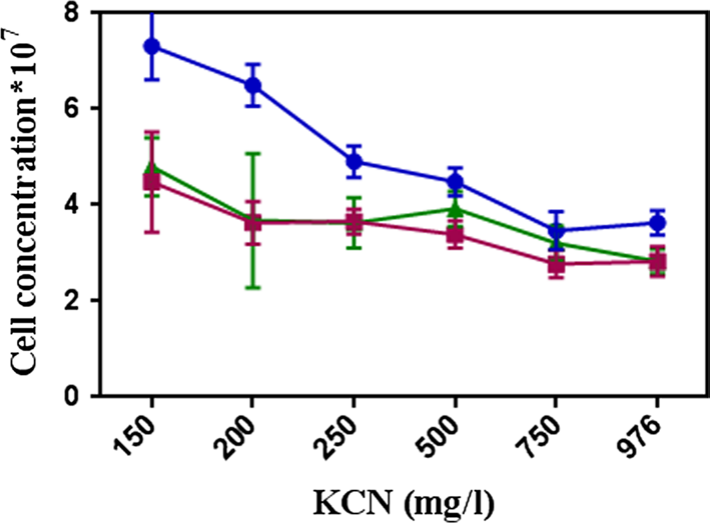
*Bacillus* sp. M01 growth in different concentration of KCN at 3 days. At different times, bacterial absorbance was measured by spectrophotometer and convert it to cell count after 24 h (square), 48 h (circle) and 72 h (triangle)

Survival of some bacterial strains in cyanide-contaminated environments implies that they may have the necessary mechanisms to tolerate and degrade cyanide compounds. In this study, cyanide-degrading bacteria were isolated from cyanide-contaminated wastewater and soil from the Muteh gold mine. Bacteria were cultured at different concentrations of KCN at 3 days in three replicates. Screening was performed among 24 isolates (data not shown), one of which was selected as the best cyanide-resistant bacterium that grows in different concentrations of cyanide within 3 days (Fig. 1).

### Identification of *Bacillus* sp. M01 PTCC 1908

In order to identify the selected bacteria, the morphological and biochemical characteristics were studied. According to Bergey’s Manual of Systematic Bacteriology (Kersters and Vancanneyt 2005), the bacteria identified as *Bacillus* sp. (Table 3) belong to the GRAS bacteria family.

**Table 3:** Biochemical and Morphological characterization of *Bacillus* sp. M01 PTCC 1908

Biochemical characterization										
Indol	Nitrate	Starch	Glucose	Arabinose	Trehalose	Lecithin	Gelatin	Casein	Salicin	Gas glucose
Negative	Positive	Positive	Positive	Negative	Positive	Negative	Positive	Positive	Positive	Negative

Morphological characterization										
Pigment	Spore	Motility	Gram	Shape						
Large	Positive	Positive	Positive	Rode shaped						

### Factors selected by Plackett–Burman design

**Fig. 2:**
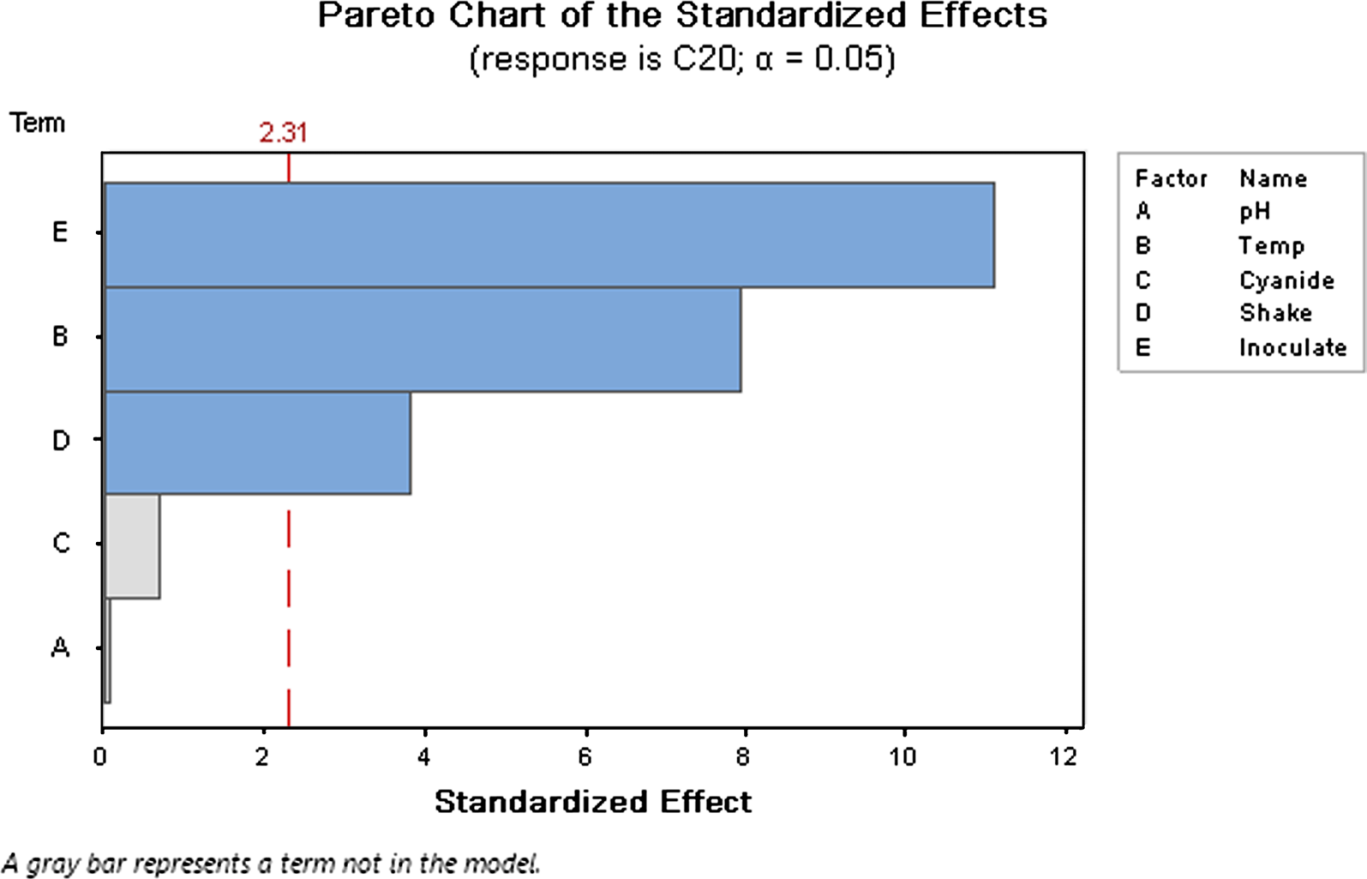
Screening the variables by Plackett–Burman design which show that the three effective factors among the all factors. The selected factors should be have P value < 0.05

The factors were screened through the Plackett–Burman design. Three factors—temperature, shaking speed, and inoculation amount—were selected among the five possible variable factors (pH, temperature, cyanide concentration, shaking speed, and inoculation amount). The P values for these three factors were less than 0.05 when α was equal to 0.05. Data analysis and related figures are shown in Table 4 and Fig. 2 respectively (R^2^ = 96%).

**Table 4:** Analysis of variance result for the parameters of Plackett–Burman design

Source	DF	Adj SS	Adj MS	F value	P value
Model	3	10,414.0	3471.34	67.12	0.000
Linear	3	10,414.0	3471.34	67.12	0.000
Temperature	1	3266.3	3266.27	63.16	0.000
Rotation speed	1	750.7	750.67	14.52	0.005
Inoculation amount	1	6397.1	6397.08	123.69	0.000
Error	8	413.7	51.72		
Total	11	10,827.8			

R^2^ = 96%, Adj R^2^ = 94.7%, Pred R^2^ = 91.4%. (α = 0.05)

*DF* degree of freedom, *SS* sum of squares, *MS* mean square

### Central composite design and data analysis

According to the model suggested by software, three variables will be selected to achieve the highest percentage of cyanide biodegradation. The factors were studied at five different levels. The full experimental plan, with regard to their values in absolute and cyanide-degrading percentage as a response is provided in Table 5. Models designed with the estimated parameters (e.g. P value and lack of fit) were determined using data analysis (ANOVA) for cyanide biodegradation (Table 6). ANOVA results indicate that the designed model is strongly significant (P value < 0.0001). Lack of fit is a very important parameter—it shows that the error does not have any effect on the model proposed by RSM. Lack of fit value is not significant if P value > 0.05. In this model, lack of fit has been obtained with P value = 0.35, which is an approved value. If this parameter becomes significant, the entire model loses significance.

**Table 5:** Experimental design and independent variables, using central composite design, and the corresponding response for cyanide biodegradation

Std. order	Temperature (°C)	Rotation speed (rpm)	Inoculation amount (%)	Response
1	30	130	1.5	52.9
2	44	130	1.5	63.5
3	30	230	1.5	84.0
4	44	230	1.5	32.2
5	30	130	5	24.0
6	44	130	5	74.2
7	30	230	5	49.3
8	44	230	5	56.4
9	25	180	3.25	92.8
10	49	180	3.25	96.1
11	37	65	3.25	69.2
12	37	264	3.25	63.1
13	37	180	0.31	32.2
14	37	180	6.19	24.7
15	37	180	3.25	59.4
16	37	180	3.25	47.7
17	37	180	3.25	49.7
18	37	180	3.25	52.7
19	37	180	3.25	61.0
20	37	180	3.25	45.2

**Table 6:** Analysis of variance result for the parameters of quadratic model by CCD of response surface methodology

Source	Sum of squares	DF	Mean square	F value	P value Prob > F	
Mode	7288.7	7	1041	19.65	< 0.0001	Significant
A: temperature	44.7	1	44.72	0.84	0.3763	Insignificant
B: rotation speed	2.75	1	2.75	0.052	0.8236	Insignificant
C: inoculation amount	107.5	1	107.5	2.03	0.1799	Insignificant
AB	1471.5	1	1471.5	27.77	0.0002	Significant
AC	1287		1287	24.29	0.0003	Significant
A^2^	2331	12	2330.9	44	< 0.0001	Significant
B^2^	119.24		119.24	2.36	0.1462	Insignificant
C^2^	1654	7	2330.9	31.22	0.0001	Significant
Residual	635.8	12	52.99			
Lack of fit	431.3	7	61.62	1.51	0.3369	Insignificant
Pure error	204.50	5	40.90			
Cor total	7809.69	19				

R^2^ = 0.91, Adj R^2^ = 0.87, PredR^2^ = 80, Adeq precision = 16.01, C.V. % = 12.57, mean = 56.53

The regression coefficient (R^2^) of the model implies that the obtained model is potentially correct. According to the analysis, our model is significant, since the obtained R^2^, AdjR^2^, and PredR^2^ are equal to 92, 87.3, and 80% respectively.

**Fig. 3:**
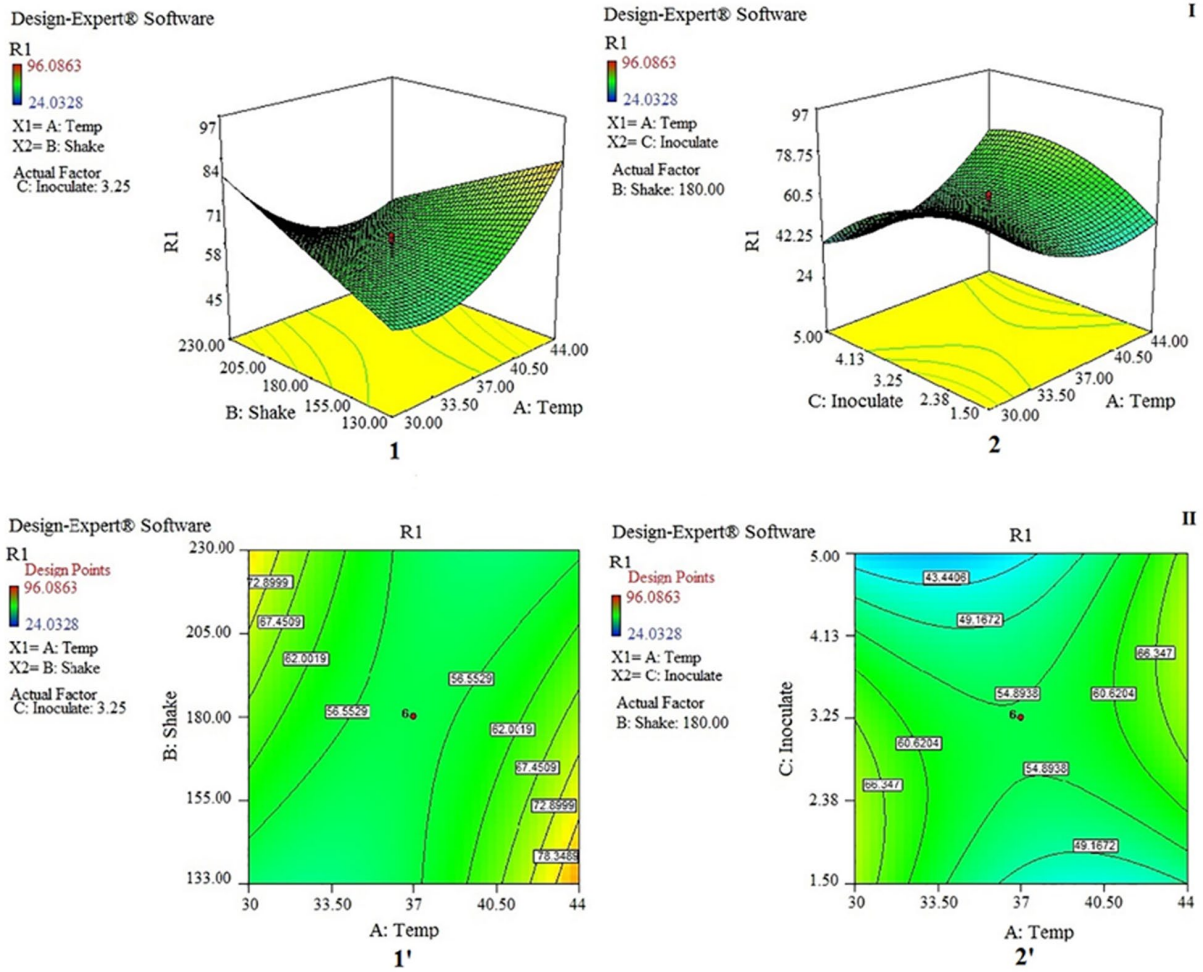
3D Response surface plots (I) and contour plot (II) between A: temperature and B: rotation speed (1 and 1′), also between A and C: inoculation amount (2 and 2′). The plots (I), showing the interaction of independent factors for cyanide degradation by *Bacillus* sp. M01. Moreover, the contour plot (II), showing the maximum rate of the cyanide degradation was shown by dark green color

In the end, after all of the software analyses, the final equation for cultural optimization process will be obtained in ANOVA, as is shown in Table 4 and Eq. 2. The 3D response surface plots and contour plot between independent factors is shown in Fig. 3.

2 $$\begin{aligned} {\text{R1}} & = + 235.81273- 15.24309 { }*{\text{ Temp }} + 1.42474 { } \hfill \\ & \quad *{\text{ Shake}} - 17.28630 * {\text{Inoculate}} - 0.038749 { } \hfill \\ & \quad *{\text{ Temp }}*{\text{ Shake }} + 1.03539 { }*{\text{ Temp }}*{\text{ Inoculate }} \hfill \\ & \quad + 0.25826 { }*{\text{ Temp}}^{ 2} - 3.48093 { }*{\text{ Inoculate}}^{ 2} \hfill \\ \end{aligned}$$

### One-sample t test and one-way ANOVA analysis

To confirm the optimal point predicted by the software, a series of experiments in five replicates were performed for determining the optimal point according to the software prediction and it was analyzed using one-sample t test. Before optimization, cyanide degradation by *Bacillus* sp. M01 PTCC 1908 was 36.7% at 10 mM initial cyanide concentration. After optimization, degradation percentage was increased to 86.4%. There was no significant difference between the predicted and experimental values, implying that the obtained data are reliable.

The model suggested for this experiment follows the second-order equation. It is very difficult to distinguish the variables with the highest influence on final result. In this experiment, the effect of temperature and shaking speed as well temperature and inoculation amount on biodegradation of cyanide is shown in Fig. 2. Therefore, cyanide biodegradation efficiency is influenced by the interaction between the variables mentioned above.

One-sample t test was employed to confirm the results obtained from RSM. For this step, the P value should be higher than 0.05. After the submission of the quadratic model data, the average of five replicates was obtained from the software. For this section, the P value was evaluated about 0.197, which means that the data follow the suggested model.

For performing one-way ANOVA test, normality and equal variance must be considered; the difference between the five replicates must be insignificant and the model designed by the software should be reliable. Therefore, the P values must be more than 0.05 (in this case they are 0.292, and 0.228 respectively).

One-way ANOVA was employed after confirmation of normality and equal variances. The result of data analysis shows that P value < 0.05 (R^2^, Adj R^2^ and R^2^Pred) > 70% are required. Our results showed that, P value = 0.000, which implies that the experiment is very significant.

**Fig. 4:**
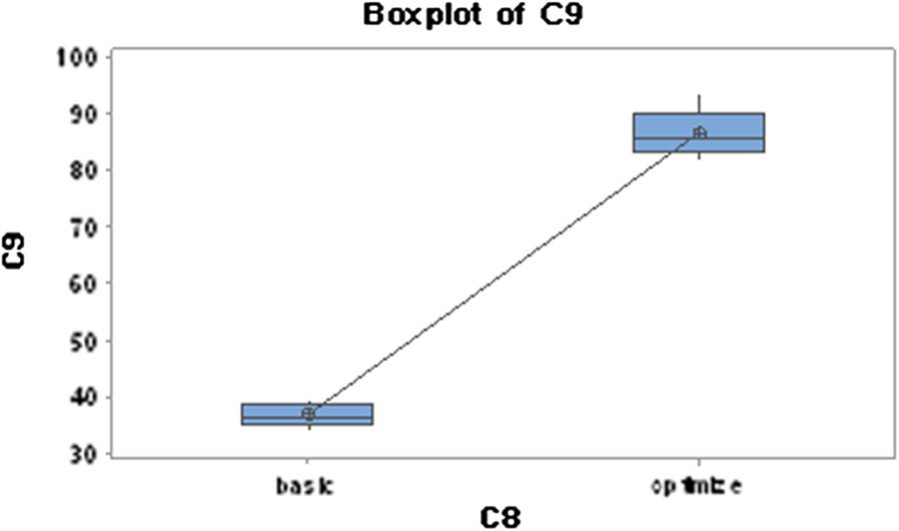
Difference assessment of cyanide degradation in basal medium (basic) and optimized medium (optimize). Basic environment, to optimize culture conditions and after obtaining the optimal environment optimized by one way ONOVA vs. t test

Also, R^2^ > 98.6, AdjR^2^ > 98.3% and R^2^Pred > 97.8, implying conformity of the suggested method and practical results. In this research, according to the results obtained from Plackett–Burman, RSM, one-way ANOVA and one-sample t test, the average percentage of cyanide biodegradation using *Bacillus* sp. M01 PTCC 1908 was increased by 2.35 times (Fig. 4).

## Discussion

In this study, the selected bacterium, with the highest tolerance to cyanide, is shown in Fig. 1 (15 mM–976 mg L^−1^), although in the expression of Soni Tiwari, microorganisms cannot tolerate a high concentration of cyanide (Tiwari et al. 2017). The maximum rate of cyanide degradation was reported to be 700 mg L^−1^ by (Wu et al. 2014). Chen et al. showed that the *K. oxytoca* could tolerate a higher CN^−^ concentration of 78 mg L^−1^ (Chen et al. 2008). Koksunan et al. reported that *A. vinelandii* could tolerate CN^−^ concentration of 104 mg L^−1^ (Sarawut et al. 2013), while the *Pseudomonas pseudoalcaligenes* W2 could tolerate a higher cyanide concentration than 39 mg L^−1^; its growth was stopped at 52 mg L^−1^ (Tiong et al. 2015). *Pseudomonas putida* could grow in 4 mM concentration of cyanide and was inhibited at 8 mM (Chapatwala et al. 1998). *Burkholderia cepacia* sp. C-3 could tolerate up to 10 mM of cyanide concentration (Adjei and Ohta 2000). An active biological process for the maximum threshold of cyanide biodegradation was observed to be 200 mg L^−1^ (Kuyucak and Akcil 2013). According to the above-mentioned data, the cyanide resistance of *Bacillus* sp. M01 PTCC 1908 is higher than that of the others.

Optimization steps were performed, and the results of this experiments showed that at the first condition, with the points suggested by the software (130 rpm, 44 °C and 4.06% inoculation amount), cyanide biodegradation percentage is estimated to be 86%, while in the second condition proposed by the software at 30 °C, 230 rpm, and 1.98% inoculation amount, percentage of cyanide biodegradation is estimated to be about 84.5%. However, in another study, the isolated microbial species were optimized through the numerical optimization technique and the optimized condition was temperature 33.60 °C, pH 9.88 and whey-waste concentration of 14.27 g L^−1^, which can biodegrade an initial cyanide concentration of 500–206.53 mg L^−1^ in 96 h (Mekuto et al. 2015). *Bacillus* sp. CN-22 can tolerate the highest CN^−^ concentration of 700 mg L^−1^. Under the optimized condition given by RSM, it can also achieve the highest degradation of CN^−^ at 96.69%, by reducing CN^−^ concentrations from 200 to 6.62 mg L^−1^ in 72 h (Wu et al. 2014). However, for biodegradation of cyanide with an initial CN^−^ concentration of 1 mg L^−1^ in 3 min, 90% can be removed. This is used for residual cyanide from wastewater treatment with low cyanide concentrations (Hijosa-Valsero et al. 2013). When equipment in the laboratory is not sufficient for conducting the experiment under optimized conditions and the implementation of this process is limited, the second condition is preferred. With regard to Eq. 2, at any time during the performance of this experiment, the result can be obtained by the replacement of the factor values in the equation without needing to perform all the steps for the biodegradation process optimization. Therefore decision can be taken with regard to whether degradation percentage is acceptable. The results of this optimization show that *Bacillus* sp. M01 PTCC 1908 can degrade 651.2 mg L^−1^ initial cyanide concentration to 562.5 mg L^−1^ in 48 h (i.e. 86.4%). A study by Kao et al. reported that cyanide concentration higher than 2.6 mg L^−1^ can induce a longer lag phase and t test result determined that the difference between growths at cyanide concentration above 13 mg L^−1^ was not significant. In another research, performed by Akcil et al., the ability of degrading weak acid dissociable cyanide (CNWAD) at 400 ppm cyanide concentration was evaluated and the efficiency was found to be nearly 90% (Akcil et al. 2003).

For removal of wastewater containing cyanide, the biodegradation method has been suggested. In this study we found that, *Bacillus* sp. M01 PTCC 1908, which has a high resistance (15 mM–976 mg L^−1^) and degrading ability (10 mM–562 mg L^−1^), was suggested for biodegradation process in many industries that dealing with this compound. In comparison with other studies, this study reported that if the optimal points of temperature of 44 °C, shaking speed of 130 rpm and inoculation amount of 4.04% are ensured, a higher maximum rate of biodegradation is observed than has ever been reported before. This process has been patented in Iranian Intellectual property centre under Licence No: 90533.

## Abbreviations

RSM: response surface methodology
ANOVA: analyze of variance
OD: optical density
PBD: Plackett–Burman design
CCD: central composite design
DF: degree of freedom
SS: sum of squares
MS: mean square
R^2^: regression coefficient
CNWAD: weak acid dissociable cyanide
